# Aluminum-induced dreierketten chain cross-links increase the mechanical properties of nanocrystalline calcium aluminosilicate hydrate

**DOI:** 10.1038/srep44032

**Published:** 2017-03-10

**Authors:** Guoqing Geng, Rupert J. Myers, Jiaqi Li, Roya Maboudian, Carlo Carraro, David A. Shapiro, Paulo J. M. Monteiro

**Affiliations:** 1 Department of Civil and Environmental Engineering, University of California, Berkeley, California 94720, United States; 2 School of Forestry & Environmental Studies, Yale University, New Haven, Connecticut 06511, United States; 3 Department of Chemical & Biomolecular Engineering, University of California, Berkeley, California 94720, United States; 4 Advanced Light Source, Lawrence Berkeley National Laboratory, Berkeley, California 94720, United States

## Abstract

The incorporation of Al and increased curing temperature promotes the crystallization and cross-linking of calcium (alumino)silicate hydrate (C-(A-)S-H), which is the primary binding phase in most contemporary concrete materials. However, the influence of Al-induced structural changes on the mechanical properties at atomistic scale is not well understood. Herein, synchrotron radiation-based high-pressure X-ray diffraction is used to quantify the influence of dreierketten chain cross-linking on the anisotropic mechanical behavior of C-(A-)S-H. We show that the ***ab***-planar stiffness is independent of dreierketten chain defects, e.g. vacancies in bridging tetrahedra sites and Al for Si substitution. The ***c***-axis of non-cross-linked C-(A-)S-H is more deformable due to the softer interlayer opening but stiffens with decreased spacing and/or increased zeolitic water and Ca^2+^ of the interlayer. Dreierketten chain cross-links act as ‘columns’ to resist compression, thus increasing the bulk modulus of C-(A-)S-H. We provide the first experimental evidence on the influence of the Al-induced atomistic configurational change on the mechanical properties of C-(A-)S-H. Our work advances the fundamental knowledge of C-(A-)S-H on the lowest level of its hierarchical structure, and thus can impact the way that innovative C-(A-)S-H-based cementitious materials are developed using a ‘bottom-up’ approach.

The atomistic structure of calcium aluminosilicate hydrate (C-A-S-H) and its similarity with the naturally occurring tobermorite mineral family have attracted significant interest in the scientific community. As a high-performance binding material in concrete, it is produced from the reaction between calcic and aluminosiliceous components in water and often under elevated temperature. This phenomena is found in the ancient Roman concrete^1^, alkali activated cements^2^,^3^,^4^ and massive-volume castings of concrete—such as in the case of dams—comprised of hydrated Portland cement (PC) blended with supplementary cementitious materials (SCMs, e.g. fly ash, blast-furnace slag, volcanic ash)^5^,^6^. Compared to Al-free calcium silicate hydrate (C-S-H), which is the most abundant and key binding phase in PC concrete), the process to produce C-A-S-H in contemporary cementitious materials usually involves incorporating more SCMs; this results in a lower CO_2_ footprint^2^, which is critical in improving the sustainability of the construction industry^7^.

C-(A-)S-H synthesized hydrothermally at room temperature lacks long-range crystallinity but has an atomistic structure analogous to tobermorite, i.e., stacked layered structures (along the ***c**-*axis), with aluminosilicate dreierketten chains clamping on either side of CaO_7_ double sheets, with water and charge-balancing ions (e.g., Ca^2+^) in the interlayer space^8^,^9^,^10^,^11^,^12^,^13^ (both defined as zeolitic contents, as shown in Fig. 1a). Every two-paired-site Si(Al)O_4_ tetrahedra are connected by a bridging site tetrahedron in the dreierketten chain structure, which runs parallel to the ***b**-*axis. The intralayer and interlayer, as separated by the bridging site Si, together lead to basal spacings of ~9–16 Å in the C-(A)-S-H structure^14^,^15^,^16^,^17^,^18^, with the thickness of the intralayer roughly constant while that of the interlayer varying greatly with the synthesizing conditions.

Unlike macro-crystalline tobermorite, C-(A-)S-H is often nano-crystalline, and contains defects such as vacancies and Al for Si substitution on the dreierketten chain. Such substitution, when occurring in the bridging site, generates the minimum energy gain, as investigated by *ab* initio calculations^16^,^17^. The preferred lengths of the aluminosilicate tetrahedra chains match the (3n-1) rule as in pure silicate chains^17^, which is also confirmed by experimental evidences^19^. The C-(A-)S-H structure can also accommodate a range of water molecules and aqueous species (e.g., Ca^2+^) in the interlayer space. Cross-linking of adjacent dreierketten chains can also occur, as shown in Fig. 1b. The long-range order of C-(A-)S-H is reported to increase with Al-incorporation and curing at elevated temperature^14^. Al-induced cross-linking is observed at 80 °C^14^ but also at lower temperatures when synthesized using aqueous alkali hydroxide solutions^14^,^20^,^21^; however, Al-incorporation typically decreases C-A-S-H crystallinity at room temperature^15^,^21^.

Despite numerous studies on the chemistry and crystallography of C-(A-)S-H, the correlation between its structure and mechanical properties at atomistic scale has rarely been experimentally studied: the influence of Al-induced cross-linking on the mechanical properties of C-(A-)S-H has never been reported. Direct experimental data on pore-free C-(A-)S-H solids are difficult to obtain because it has hierarchical porosity down to the atomistic scale. Although laboratory methods such as nano-indentation measure the elastic modulus and hardness of materials on the order of several μm, they only provide indirect results based on statistical averaging and homogenization assumptions^22^,^23^. On the other hand, synchrotron radiation-based high-pressure XRD (HP-XRD) allows probing crystal lattice deformations under hydrostatic pressure which intrinsically eliminates the influence of nano-porosity, since the pressure medium efficiently penetrates into the nano-pores and generates uniform pressure on the solid. A recent HP-XRD study reported the bulk modulus of macro-crystalline Al-tobermorite to be 55 ± 5 GPa^24^. It was also previously reported that Al-incorporation does not change the mechanical properties of nano-crystalline C-A-S-H in alkali-activated slag (AAS) cement (38 ± 3 GPa) relative to C-S-H(I) (38 ± 7 GPa)^25^; however, the differences in the sample preparation conditions employed for each material and the lack of chemical crystallographic characterization shed doubt on the reliability of this conclusion. Therefore, the influence of Al incorporation on the atomistic scale mechanical properties of C-A-S-H remains unclear. This information is critical for understanding, predicting and designing the mechanical performance of concrete structures comprised of C-(A-)S-H-based composites, i.e., the great majority of contemporary construction materials.

Atomistic simulations, bypassing the experimental difficulties, provide feasible probes of atomistic structures and properties. Starting from defective tobermorite structures^26^,^27^,^28^,^29^,^30^,^31^, simulations of C-(A-)S-H have been reported, however there exist clear missing gaps that restrict its improvement: (a) a lack of direct experimental tests to validate the simulated mechanical properties at the atomistic scale; (b) to date, focus has been directed on the structural change but limited work has been performed on its influence to the mechanical properties^30^,^31^; and (c) the published large volume of classic molecular dynamics study may not provide information as accurate as ab initio calculation does, in terms of investigating the interatomic bonding, especially in the interlayer space^32^,^33^.

Reported herein for the first time, we used samples synthesized at 80 °C to experimentally assess the influence of Al-induced crystallographic changes (e.g. dreierketten chain cross-linking) on the mechanical properties of synthetic nano-crystalline C-(A-)S-H. Their lattice-axial and volumetric responses to hydrostatic loading (up to ~10 GPa) were determined by synchrotron radiation-based HP-XRD, which were then used to calculate the bulk moduli values. X-ray ptychography was conducted at a pixel resolution of ~5 nm to directly image the C-(A-)S-H nano-morphology. The results of this work provides fundamental information for understanding the structure and mechanical behavior of C-(A-)S-H at the lowest level of its hierarchical structure, which is essential to establish a predictive multi-scale model of modern cementitious materials.

## Results and Discussion

### XRD and ptychography at ambient pressure

**Figure 2:**
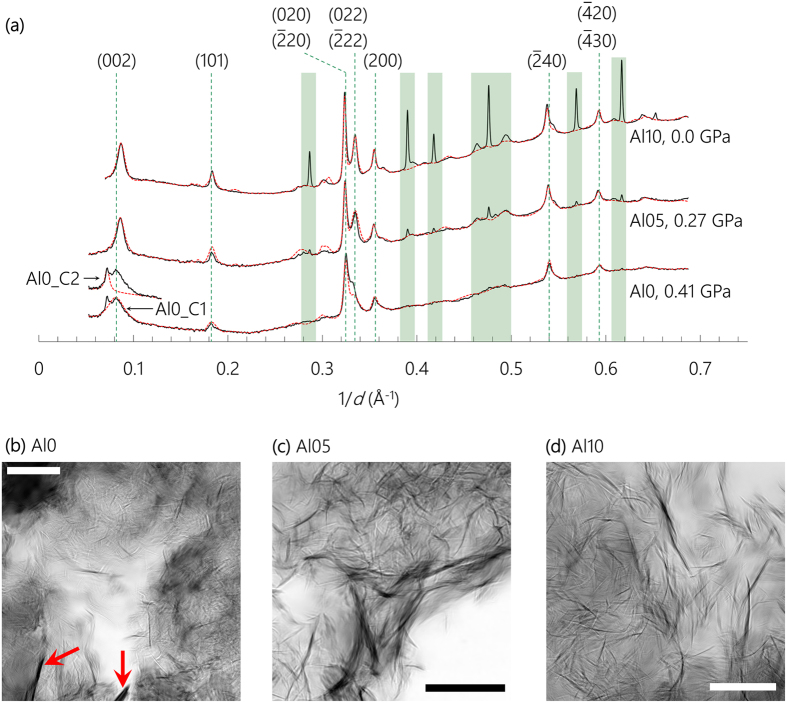
(**a**) Reference X-ray diffractograms (solid black curves) of Al0, Al05, and Al10 at 0.41, 0.27, and 0.0 GPa, respectively. Calculated diffractograms from Rietveld refinement are displayed as red curves. Typical diffraction peaks of C-(A-)S-H are assigned to their Miller indices (dashed vertical green lines). Diffraction from ruby and steel gaskets are masked by green strips. X-ray ptychographic images of (**b**) Al0, (**c**) Al05, and (**d**) Al10. Scale bars are 1 μm. The Al0_C2 diffraction may come from the relatively large crystalline regions in (**b**), labeled by red arrows, whereas Al0_C1 is assigned to the more abundant thin fibers.

C-(A-)S-H samples studied here are of the same Ca to Si molar ratio (Ca/Si) = 1.0 but with different Al to Si molar ratios (Al/Si) = 0.0, 0.05, 0.1, and are labeled Al0, Al05 and Al10, respectively. Cross-linking of adjacent layers was confirmed to exist in Al10, and not exist in Al0, according to published study on the same sample^14^. The average number of AlO_4_ and SiO_4_ tetrahedra in the dreierketten chains of the Al0 and Al10 samples are 8.8 and 19.8, respectively^14^. For further detail, see the Method section. The XRD diffractograms obtained at the lowest probed pressure, (0.41, 0.27, and 0.0 GPa for Al0, Al05, and Al10, respectively), are used as ambient condition references (Fig. 2a). The diffraction peaks, although clearly sharper here, generally match those present in typical laboratory XRD patterns of C-S-H(I): peaks with 1/*d* > 0.3 Å^−1^ are assigned to (*hk0*) reflections^34^,^35^,^36^.

In general, the crystallographic information contained in the diffractograms here are insufficient to enable refinements of atoms positions, due to the limited number of and relatively broad diffraction peaks. By adjusting the lattice parameters, satisfactory fitting can be obtained using a few different tobermorite models, as well as defective tobermorite models suggested in reported work^13^, although these models clearly differ in basal spacing and Ca/Si. A detailed comparison among refinements with different models is available in Supporting Information (SI) file. In order to keep the analysis consistent across all samples, all diffraction patterns are refined starting from the same 11 Å tobermorite structure (monoclinic, space group *b11m*)^12^. It should be noticed that it is possible to vary the refined basal spacing between 9 Å and 14 Å, even though the refining starts from a 11 Å tobermorite model. The refinement is then conducted by sequentially modifying the three most significant structural characteristics: (1) the anisotropic nano-crystallite sizes; (2) the micro-strain along ***c***-axis due to disordered stacking; and (3) adjusting the lattice parameters ***a**, **b**, **c***, and angle *γ*. The anisotropic crystallite sizes are estimated from the line broadening model “anisotropic – no rules” as described in the literature^37^. These crystallographic features are readily defined in the *MAUD* software package^38^.

As shown in Fig. 2a, a peak near 1/*d*~0.07–0.09 Å^−1^ is observed for all samples and is assigned to (002) reflections. In Al0, a much sharper second basal peak is observed at ~0.07 Å^−1^—other than the major basal peak observed at ~0.08 Å^−1^—indicating two major nano-crystalline structures in this sample that share similar structures in ***ab***-plane but have different basal spacings; they are hereafter named Al0_C2 and Al0_C1, respectively. Refinement of the Al0_C1/Al0_C2 mass ratio yields a value of 5. Weak diffraction of (101) is observed for all samples. The (022) and (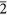
22) diffractions at ~0.335 Å^−1^ are clearly observed in Al10 and Al05 but less resolvable in Al0. The relative intensities and sharpness of the peaks associated with the ***c***-axis indicate that stacking along this axis is more ordered in Al10 and Al05 than in Al0_C1. This verifies that Al-incorporation increases the crystallinity of C-A-S-H in these 80 °C samples, which is consistent with the laboratory X-ray diffraction results of these materials^14^.

The refinement results presented in Table 1 show that Al-incorporation increases the lattice parameters ***a*** and ***b*** since the typical AlO_4_ tetrahedron (Al-O ≈ 1.75 Å in katoite (Ca_3_Al_2_O_6_∙10H_2_O), for instance^39^) is larger than the SiO_4_ tetrahedron (Si-O ≈ 1.62 Å in 11 Å tobermorite^12^). The basal spacing, however, is significantly reduced by Al-induced cross-linking in Al10 (11.5 Å)^14^, which most likely also occurs in Al05 (11.6 Å) due to the similarly significantly reduced basal spacing with respect to Al0_C1 (12.2 Å) and Al0_C2 (13.8 Å). The sharpness and relative intensities of the (002) peaks are highly sensitive to the crystallite size along the ***c***-axis (*l*_*cc*_); therefore, refining *l*_*cc*_ yields uncertainties of 0.5–1 nm, while the other dimensions (*l*_*aa*_ and *l*_*bb*_) with uncertainties of 5 nm. The Al0_C2 has a much larger crystallite size along the ***c***-axis than Al0_C1 (15 and 3.5 nm, respectively). Compared with Al0_C1, Al-incorporation has at least doubled the size of nano-crystals along the ***c***-axis in Al05 and Al10. The refinement of lattice parameter ***a*** is largely determined by the positions of the (200) peaks, whereas refining ***b*** mainly depends on the positions of the (020) & (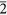
20) peaks in addition to ***a***. Both lattice parameter values have uncertainties of ± 0.01 Å. Refinement of ***c*** is essentially independent of ***a*** and ***b***, and is mainly determined by the (002) peak position (uncertainty of ± 0.1 Å).

Nano-scale morphologies of the samples are illustrated by the X-ray ptychographic images as shown in Fig. 2b–d. All samples exhibit agglomerated layered/fibrillar structure at nano-scale. The fibrillar precipitates are clearly thicker and longer in Al05 and Al10 than in Al0. Apart from the majority of areas in Fig. 2b that are composed of thin fibers, relatively thick rods are also identified as indicated by red arrows. These rods exhibit sharp edge and are most probably well-crystalline materials corresponding to the Al0_C2 phase from the refinement. Consequently, the thin and short fibers in Fig. 2b are assigned to Al0_1 phase. We provide a quantitative analysis in the SI file, to support the direct observation here, that the thickness of the poorly-crystalline C-S-H layer structure (Al0_C1) is increased by incorporating Al, i.e. from ~9 nm in Al0_C1 to ~19 and 13 nm in Al05 and Al10, respectively. This is consistent with the refinement results where the crystallite sizes of Al05 and Al10 are larger than that of Al0_C1.

### High-pressure XRD

**Figure 3:**
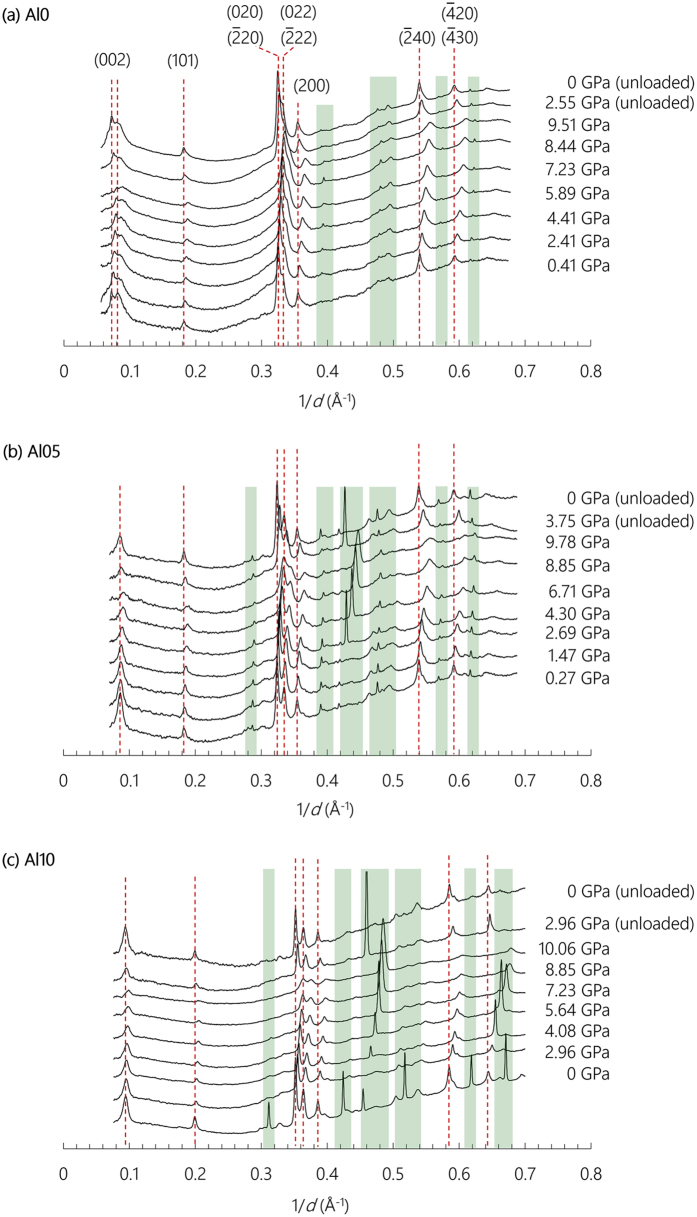
Normalized XRD diffractograms of Al0, Al05, and Al10 up to ~10 GPa. Vertical red dashed lines correspond to the reference C-(A-)S-H peaks positions at ambient pressure as eye guides. Two XRD patterns taken after unloading pressure are plotted for each sample. Diffraction from ruby and steel gaskets are masked by green strips. The determination of pressure has uncertainty of ~0.15 GPa.

When samples are exposed to increasing hydrostatic loading, all resolvable diffraction peaks of nano-crystalline C-(A-)S-H shift to higher 1/*d* values independent of the Al content (Fig. 3). The deformations are purely elastic as these peaks return to their original positions upon unloading. Each individual peak broadens with increasing pressure, which is caused by reduced crystallinity and/or the development of micro-strain and are common phenomena observed in HP-XRD experiments^40^. The monotonically increasing peak shifting and the absence of peak splitting or the occurrence of new peaks imply no reconstructive crystal-structural change during the loading process. However, peak broadening does cause some adjacent peaks to merge, e.g., the (020) & (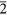
20) peaks with the (022) & (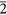
22) peaks in Al05 and Al10 at pressure >~8 GPa.

**Figure 4:**
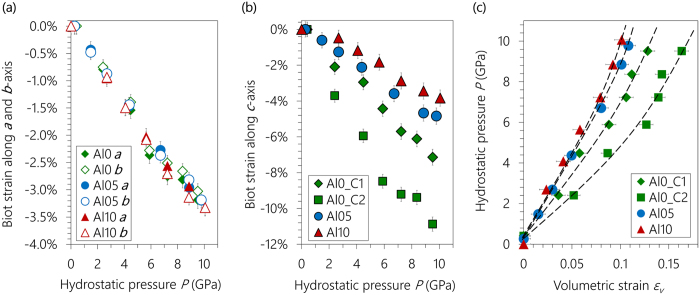
Biot strains as functions of applied hydrostatic pressure along (**a**) ***a***- and ***b**-*axes, and (**b**) ***c**-*axis. (**c**) Hydrostatic pressure as a function of volumetric strain. Biot strain is defined as *ε* = *L/L*_*0*_ − 1, where *L* and *L*_*0*_ are the current and initial length of unit cell edge, respectively. The error bars of the axial Biot strains and the volumetric strain originate from the refinement uncertainties (see Table 1). The uncertainty of pressure is estimated to be ~0.15 GPa. Dashed curves in (**c**) illustrate the fitted results of BM-EoS.

The refinement protocol described above for the results shown in Fig. 2 is used to fit the diffraction patterns shown in Fig. 3, the results of which are plotted in the form of Biot strains (*ε* = *L/L*_*0*_ − 1, where *L* and *L*_*0*_ are the current and initial length of unit cell edge, respectively) in Fig. 4a,b. In a HP-XRD study of 14 Å tobermorite^41^, the angle *γ* was found to fluctuate within ±0.1° with hydrostatic pressure increased to ~5 GPa, which induces a volumetric strain (*ε*_*V*_ = 1 − *V/V*_*0*_, where *V* and *V*_*0*_ are the observed and initial unit cell volume) of ±0.001 and is negligible in determining bulk modulus. Hence, *γ* was fixed to its respective values in each sample at ambient condition (Table 1), leaving only ***a**, **b*** and ***c*** to be refined.

The stiffness of the nano-crystalline C-(A-)S-H along the ***a***- and ***b***-axes are comparable and independent of their Al content (Fig. 4a). These axes deform monotonically as functions of pressure up to ~10 GPa, with average compliance (defined as the absolute value of the slope of the *ε*-*P* plot) ≈ 3.3% per 10 GPa. Similar compliances have been reported for synthetic C-S-H(I)^25^ and 14 Å tobermorite^41^, which implies that the ***ab***-planar stiffness of C-(A-)S-H is not significantly influenced by the extent of bridging site vacancies, nor Al for Si substitution in their dreierketten chains.

Deformation along the ***c***-axis in Al0 is roughly linear, with compliance ≈7% per 10 GPa for Al0_C1, and ≈11% per 10 GPa for Al0_C2 (Fig. 4b). The material is clearly softer along the ***c***-axis than within the ***ab**-*plane. The compliance of the ***c***-axis in Al0_C2 is consistent with reported data for that found in 14 Å tobermorite macro-crystal^41^. As its ambient lattice parameter ***c*** (27.6 Å) is also consistent with that of 14 Å tobermorite (27.99 Å^8^), Al0_C2 is most probably nano-crystalline 14 Å tobermorite. This conclusion collaborates existing work which demonstrates that 14 Å tobermorite is often found intermixed with normal 11 Å tobermorite in the absence of Al (i.e., Al0_C2 and Al0_C1, respectively) but not in the presence of Al (i.e. the case of Al05 and Al10)^42^. Compared with Al0, the ***c***-axis is clearly stiffer in the Al-containing samples, which stiffens as the Al content increases. The compliance along the ***c***-axis of Al10 (~4% per 10 GPa) is almost comparable to those along the ***a***- and ***b***-axes.

**Figure 5:**
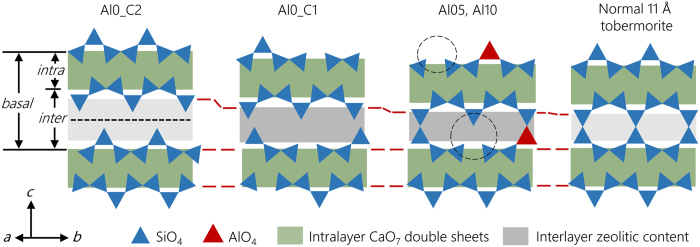
Schematic comparison of atomistic configurations of C-(A-)S-H in the samples analyzed here and in normal 11 Å tobermorite. The darkness of the grey band in the interlayer illustrates the concentration of the zeolitic content (e.g., interlayer water and Ca^2+^, dark = more concentrated and light = less concentrated). A ***b***/2 glide plan (black short-dashed line) is assumed in Al0_C2 following 14 Å tobermorite^8^. The black dashed circles indicate non-cross-linked sites due to missing bridging tetrahedra in C-A-S-H samples. The red long-dashed lines are eye guides to view the changing basal and interlayer spacing. The terms *basal, inter*, and *intra* represent basal, interlayer, and intralayer spacing, respectively. The amount of vacancies in Al0_C1 and Al_10 were roughly determined using equations in published work^13^,^51^.

The bulk modulus *K*_*0*_ under ambient pressure is fitted from Fig. 4c using Birch-Murnaghan equation of state (BM-EoS, see methods, Equations (1) and (2)). Note that *K*_*0*_*′* is often suggested as having a constant value of 4 for zeolites^40^,^41^,^42^,^43^, thus, leaving *K*_*0*_ as the only fitted parameter in Equation (2). The results of these fits are shown in Table 2. The *K*_*0*_ of Al0_C2 (36 ± 1 GPa) is comparable to the reported value for macro-crystalline 14 Å tobermorite (47 ± 4 GPa), further supporting the interpretation herein that this material may be nano-crystalline 14 Å tobermorite^41^. The Al0_C1 has a significantly smaller basal spacing than Al0_C2 (12.2 Å versus 13.8 Å shown in Fig. 5), which makes its *c*-axis less deformable and causes its ambient pressure bulk modulus to increase by ~40%, to 50 ± 2 GPa. The presence of similar intralayer structures and the absence of cross-linking in both materials leads to the hypothesis that the interlayer, which is filled with zeolitic contents (i.e., mainly water and Ca^2+^), is much softer than the intralayer and therefore originates most of the deformation along the ***c***-axis.

Al-incorporation further reduces the basal spacings of C-A-S-H to 11.6 Å (Al05) and 11.5 Å (Al10). Although this additional reduction is less than that occurs from Al0_C2 to Al0_C1, the ambient pressure bulk modulus significantly increases to 64 ± 3 (Al05) and 71 ± 3 GPa (Al10). Therefore, the nano-crystalline C-(A-)S-H analyzed here becomes stiffer with increasing Al incorporation. A contradictory result was obtained for C-A-S-H in alkali activated slag (AAS), where no Al-induced stiffening was observed compared to C-S-H(I) synthesized at room temperature^25^. In 11 Å tobermorite the adjacent layers are cross-linked by Q^3^ SiO_4_ tetrahedra, resulting in a basal spacing ~11.24 Å^12^. As restricted by the Si-O bond length and the cross-linked chain configuration, the basal spacing of any cross-linked C-(A-)S-H should be consistent with that of 11 Å tobermorite. Note that the basal spacing of the AAS sample is 12.44 Å at ambient pressure, which is ~1.2 Å wider than that of 11 Å tobermorite. This large extra opening clearly indicates that the AAS sample is not cross-linked. Therefore, cross-linking appears to be the key factor that significantly increases the stiffness of the C-A-S-H samples studied herein. As shown in Fig. 5, the cross-linked tetrahedra between adjacent dreierketten chains may act as supporting columns that withstand the closing of the porous interlayer. This hypothesis is confirmed by simulation models which predicts that, in the interlayer region the strong Si-O bond order is predominant in 9 Å and 11 Å tobermorite, compared to jennite and 14 Å tobermorite^32^.

Cross-linked normal and anomalous 11 Å tobermorite structures^12^ are reported to have bulk moduli of 71(4) GPa (normal) and 63(2) GPa (anomalous)^44^, which is within the range defined by the moduli of Al05 and Al10. The difference between normal and anomalous 11 Å tobermorite may be attributed to different concentrations of zeolitic content (i.e., Ca^2+^ and water) in their interlayer space: Ca_zeolitic_/Si = 0.08 and 0 in normal and anomalous, respectively, and H_2_O_zeolitic_/Si = 0.83 for both^12^. The presence of zeolitic contents would enhance the steric constraints of the interlayer, thus increasing the bulk modulus. As shown in an *ab* initio work^33^, the zeolitic contents can have significant impact on the mechanical property of C-(A-)S-H, and they contribute mainly through the hydrogen bond and Ca-O bond. As a comparison, Al05 and Al10 studied herein have more zeolitic contents (Ca_zeolitic_/(Si + Al) = 0.25–0.3, and H_2_O_zeolitic_/Si = 1.1–1.2)^14^, but their bulk moduli are not higher than normal 11 Å tobermorite because the C-A-S-H samples contain non-cross-linked sites. They are generated by the omission of bridging tetrahedral, as indicated by the black dashed lines in Fig. 5.

Atomistic simulations of defective tobermorite with Ca/Si ≈ 1.0 yield results (53–62 GPa^26^ and 55–65 GPa^27^) that are generally consistent with the bulk modulus of Al0_C1 measured herein (50 ± 2 GPa). Calculated values of the bulk modulus of crystalline 14 Å tobermorite (35.91 GPa^28^ and 46 GPa^29^) are also consistent with the measured value of Al0_C2 here (36 ± 1 GPa). Therefore, we conclude that the current computational simulations provide reliable predictions of C-S-H. However, the Al-incorporated structure (C-A-S-H) has not been modelled as much as C-S-H. Our results also suggest that the zeolitic water and cations clearly contribute to the mechanical response along the *c*-axis. This is also concluded in the thermal dehydration study of 11 Å tobermorite where the interlayer Ca may cause significant rearrangement of the interlayer configuration and lead to a collapse of the interlayer in ‘normal’ 11 Å tobermorite^12^. Such special role of the zeolitic contents is not completely addressed in molecular dynamics studies, but are included in some first principle studies^32^,^33^. Both the effect of Al-incorporation and zeolitic contents need to be studied further to fully understand the structure-property correlation in C-(A-)S-H. Meanwhile, our results here provide experimental evidence to validate such simulations on C-(A-)S-H. It is reasonable to conclude that this ‘bottom-up’ approach to simulate and optimize the mechanical properties of C-(A-)S-H-based cementitious materials is promising given accurate atomistic representations of the C-(A-)S-H phase, which can be derived from experimental results discussed here.

## Conclusion

This paper reports on an analysis of the mechanical behavior of nano-crystalline C-(A-)S-H under hydrostatic load with synchrotron radiation-based HP-XRD. The results are supported by ptychographic imaging. These experiments test the lattice deformation of nano-crystalline C-(A-)S-H and for the first time systematically correlates its atomistic structures and mechanical properties. The work contributes fundamental knowledge to the understanding of the morphology and structure of nano-crystalline C-(A-)S-H, which can be used to build and validate more realistic simulation models of this material at atomistic- and nano-scales. Highlights are summarized as follows:

1. Through Rietveld refinement, we verify that Al-incorporation increases the crystallinity of C-(A-)S-H, especially along the ***c***-axis. Al-induced cross-linking is observed in C-A-S-H synthesized at 80 °C, which has a similar basal spacing to 11 Å tobermorite. The cross-linked AlO_4_ and SiO_4_ tetrahedra act as supporting ‘columns’ along the ***c**-*axis that resist closing of the interlayer space subjected to hydrostatic compression, thus increasing the overall bulk modulus. The overall bulk modulus is also correlated to the concentration of the zeolitic contents (i.e., water and Ca^2+^) and negatively correlated to the interlayer spacing.
2. The ***ab***-planar deformation under hydrostatic pressure reported herein is highly reproducible throughout all of the reported HP-XRD studies of C-(A-)S-H and tobermorite crystals synthesized in distinct conditions, leading to the conclusion that the dreierketten chain defects, e.g., bridging site vacancies and Si for Al substitution, do not significantly change the stiffness of these materials along the ***a**-* and ***b***-axes.
3. Atomistic simulations of C-S-H using defective tobermorite structures as a starting point yield generally comparable bulk moduli values to those determined here. The effect of Al-incorporation to the mechanical property of C-(A-)S-H discovered here may help to improve current simulation models to better serve the purpose of designing innovative C-(A-)S-H-based cementitious materials using a ‘bottom-up’ approach.

## Methods

### Sample preparation

Synthesis of C-(A-)S-H was performed using an initial bulk Ca/Si molar ratio of 1.0 and initial bulk Al/Si molar ratios of 0, 0.05 and 0.1 (labeled Al0, Al05, and Al10, respectively) by mixing stoichiometric amounts of SiO_2_ (Aerosil 200, Evonik), CaO (obtained by burning CaCO_3_ (Merck Millipore) at 1000 °C for 12 hours) and CaO·Al_2_O_3_ at a water/solid ratio of 45 in a N_2_-filled glove box. The mixtures were stored in Teflon bottles at 80 °C and shaken twice a week for 56 days. The solid C-(A-)S-H were vacuum filtered using 0.45 μm nylon filters, freeze-dried for 7 days, and then stored in N2-filled desiccators in the presence of saturated CaCl_2_ solutions (≈30% R.H.) and NaOH (CO_2_ trap). The average number of AlO_4_ and SiO_4_ tetrahedra in the dreierketten chains of the Al0 and Al10 samples are 8.8 and 19.8, respectively^14^.

### High-pressure X-ray diffraction (HP-XRD)

The HP-XRD experiment was conducted at beamline 12.2.2 of the Advanced Light Source (ALS) at Lawrence Berkeley National Laboratory (LBNL), using a Merrill-Bassett diamond anvil cell. Stainless steel gaskets were pre-indented by the diamond anvil (culet diameter ~300 μm) to thickness ~100 μm, and then a cylindrical chamber of diameter 150 μm was produced at the center of the indentations by laser drilling. Small quantities of ruby powder (*α*-Al_2_O_3_ doped with 0.05 wt.% Cr^3+^) were mixed with C-(A-)S-H and then loaded into the chamber, which was then filled with the pressure medium (methanol:ethanol = 4:1 by volume) that is able to penetrate into pore of size ~1 nm^45^. Immediately afterwards the diamond anvil pairs were closed. Hydrostatic pressure was generated by applying axial (i.e., parallel to the incident X-ray beam) load on the cell frame. The pressure value was measured using the ruby florescence calibration method^46^. For each sample, the experiment included 7–8 values of pressure up to ~10 GPa. Diffraction patterns were recorded on a *MAR345* image plate detector. The 2D raw image data were integrated to diffractograms using *Dioptas* software^47^ after calibrating the center positions, sample-to-detector distance, and wavelength with LaB_6_. The incident beam energy was set to ~25 keV, and the wavelength was calibrated as 0.49755 Å. Lattice parameters were refined using *MAUD* software^38^.

The pressure-induced volumetric change is related to the bulk modulus by the third-order Birch-Murnaghan equation of state (BM-EoS)^48^:


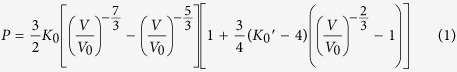


where *V*_*0*_ is the initial unit cell volume at ambient pressure; *V* is the unit cell volume at pressure *P*; *K*_*0*_, and *K*_*0*_*′* are the bulk modulus and its pressure derivative, respectively, at ambient pressure. The *K*_*0*_*′* is often suggested as a constant value of 4 for zeolites^40^,^41^,^42^,^43^; Equation (1) is then simplified to


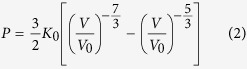


The unit cell volume *V* is calculated with the refined lattice parameters at each pressure. For a monoclinic structure, *V* is calculated as:


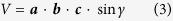


where ***a**, **b***, and ***c*** are the unit cell edge lengths, and *γ* is the angle between non-orthogonal cell edges ***a*** and ***b***.

### X-ray Ptychography

Ptychographic images were collected at beamline 5.3.2.1 in ALS of LBNL with a typical scanning transmission X-ray microscope set-up as described in literature^49^. The C-(A-)S-H samples were spread onto Si_3_N_4_ windows of thickness ~100 nm and then enclosed using a second Si_3_N_4_ window. The rims of the two windows were then sealed together with superglue, which created air-tight chambers in between. Scattering signals of the C-(A-)S-H materials were collected and reconstructed to magnitude-contrast images. The use of soft X-ray (~800 eV) and the non-damaging sample preparation process helps to preserve its nano-scale morphology. Soft X-ray ptychography provides the highest resolution ever reported for an X-ray microscope and is a state-of-the-art imaging tool to study materials that are vulnerable to electron and hard X-ray beam damage^50^.

## Additional Information

**How to cite this article**: Geng, G. *et al*. Aluminum-induced dreierketten chain cross-links increase the mechanical properties of nanocrystalline calcium aluminosilicate hydrate. *Sci. Rep.* **7**, 44032; doi: 10.1038/srep44032 (2017).

**Publisher's note:** Springer Nature remains neutral with regard to jurisdictional claims in published maps and institutional affiliations.

## Supplementary Material

Supporting Information

## Figures and Tables

**Figure 1:**
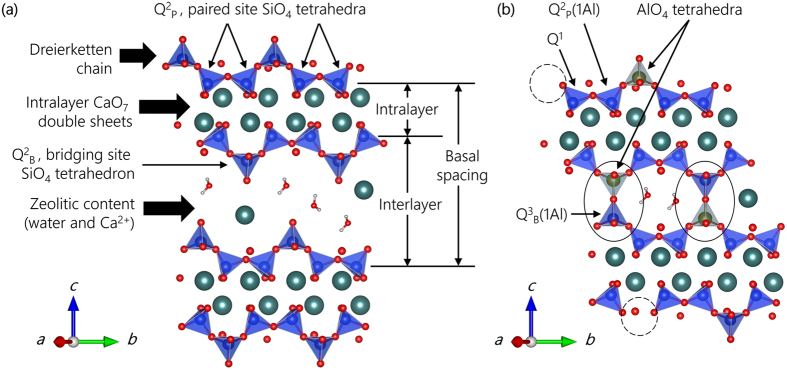
Schematics of (**a**) crystalline C-S-H (modified from 14 Å tobermorite^8^) and (**b**) C-A-S-H atomistic structures with Al-induced cross-linking (solid circles). Spheres of blue, red, green, white and yellow colors represent Si, O, Ca, H and Al, respectively. The dashed circles are tetrahedral Si vacancies in bridging sites (**b**). The conventional Q^*n*^(*m*Al)-notation is used, which describes aluminosilicate chain polymerization, e.g., Si tetrahedra are connected to *n* adjacent tetrahedra (SiO_4_ or AlO_4_), of which *m* are AlO_4_; subscripts P and B represent paired and bridging sites, respectively.

**Table 1:** Refinement of the lattice parameters (*a, b, c* and *γ*) and crystallite sizes (*l*_*xx*_, with *x* being *a, b*, or *c*) at ambient conditions.

	Al0	Al05	Al10
***a*** (Å)	6.68 ± 0.01	6.69 ± 0.01	6.71 ± 0.01
***b*** (Å)	7.33 ± 0.01	7.35 ± 0.01	7.36 ± 0.01
***c*** (Å)	24.4 ± 0.1 (Al0_C1)	23.1 ± 0.1	22.9 ± 0.1
	27.6 ± 0.1 (Al0_C2)		
*γ*	123.0° ± 0.1°	123.0° ± 0.1°	123.2° ± 0.1°
*l*_*aa*_ (nm)	18 ± 5	25 ± 5	25 ± 5
*l*_*bb*_ (nm)	25 ± 5	45 ± 5	45 ± 5
*l*_*cc*_ (nm)	3.5 ± 0.5 (Al0_C1)	7 ± 1	7.5 ± 1
	15 ± 1 (Al0_C2)		
*l*_*ab*_ (nm)	25 ± 5	45 ± 5	45 ± 5
*l*_*bc*_ (nm)	13 ± 5	20 ± 5	20 ± 5
*l*_*ac*_ (nm)	13 ± 5	20 ± 5	20 ± 5

The uncertainties of the lattice parameters are determined as 0.01 Å for ***a*** and ***b***, 0.1 Å for ***c***, and 0.1° for angle *γ*.

**Table 2:** Fitted *K*_*0*_ values from the refined lattice parameters with BM-EoS equation.

	*K*_*0*_ (GPa)	*K*_*0*_’
Al0_C1	50 ± 2	4 (assumed)
Al0_C2	36 ± 1	4 (assumed)
Al05	64 ± 3	4 (assumed)
Al10	71 ± 3	4 (assumed)

Linear fitting with two-dimensional errors follows the York method^52^.
