# Supplementary material for: Aluminum-induced dreierketten chain cross-links increase the mechanical properties of nanocrystalline calcium aluminosilicate hydrate
Source: Sci Rep. 2017 Mar 10;7:44032. doi: 10.1038/srep44032 (PMC5345016; doi:10.1038/srep44032)
Supplement: Supporting Information [file srep44032-s1.pdf]

Supporting information for:

Aluminum-induced dreierketten chain cross-links increase the mechanical properties of nanocrystalline calcium aluminosilicate hydrate

Guoqing Geng [1,*], Rupert J. Myers [1,2], Jiaqi Li [1], Roya Maboudian [3], Carlo Carraro [3], David A. Shapiro [4], Paulo J. Monteiro [1,4,*]

[1] Department of Civil and Environmental Engineering, University of California, Berkeley, California 94720, United States

[2] School of Forestry & Environmental Studies, Yale University, New Haven, Connecticut 06511, United States

[3] Department of Chemical & Biomolecular Engineering, University of California, Berkeley, California 94720, United States

[4] Advanced Light Source, Lawrence Berkeley National Laboratory, Berkeley, California 94720, United States

[*] Correspondence and request should be addressed to G.G. (guoqinggeng1989@gmail.com) or P.J.M. (monteiro@berkeley.edu)

Quantitative analysis of the ptychographic images.

Theoretical calculation of small angle scattering (SAS) from 2D transmission image has been proven useful to yield quantitative morphological information.[1] For a transmission image with intensity P at the point (x, y), the attenuation U is[2]:

$$U(x,y) = -ln[P(x,y)/P_0] = \int_{-\infty}^{+\infty} \mu(x,y,z)dz \quad (1)$$

where P_0 is the intensity of the background, and μ is the attenuation coefficient. Taking the Fourier transform of U gives:

$$FT[U](q_x,q_y) = \int_{-\infty}^{+\infty}\int_{-\infty}^{+\infty}\left[\int_{-\infty}^{+\infty} \mu(x,y,z)dz\right]\exp\left(-i\left[q_x x + q_y y\right]\right)dxdy \quad (2)$$

On the other hand the small angle scattering spectrum I is:

$$I(\boldsymbol{q}) = \frac{1}{V_0}|A(\boldsymbol{q})|^2 \text{ with } A(\boldsymbol{q}) = \iiint \rho(\boldsymbol{r})\exp(-i\boldsymbol{qr}). \quad (3)$$

with $\boldsymbol{q}$ being the scattering vector and ρ the density of scattering objects. Assuming that $\mu(x,y,z) = K\ \rho(x,y,z)$ with K a constant,

$$A(q_x,q_y,q_z = 0) = K \cdot FT[U](q_x,q_y) \quad (4)$$

Considering an isotropic material, $\rho(\boldsymbol{r}) = \rho(r)$ with $r = |\boldsymbol{r}|$, hence:

$$A(\boldsymbol{q}) = A(q) = K \cdot FT[U](q) \left(= \frac{K}{2\pi}\int_0^{2\pi} FT[U](qcos\theta, qsin\theta)d\theta\right) \quad (5)$$

And therefore:

$$I(q) = \frac{1}{V_0}K^2|FT[U](q)|^2 = \frac{1}{V_0}K^2\left|FT\left[-ln[P/P_0]\right](q)\right|^2 \quad (6)$$

This algorithm is embedded in *ImageJ™* as a plug-in. Quantification of the nanoscale morphological information of the studied C-(A-)S-H samples is conducted by applying the SAS calculation on the selected regions of the ptychographic images (Supplementary Figure 1a-c, red squares). The calculated SAS are plotted in Supplementary Figure 1d-f (black squares). To analyze the calculated SAS, we follow a recently proposed Guinier-Porod model which considers

contributions from a low-q Guinier region, an intermediate-q Guinier region and a linear Porod region. Adjacent two regions intersect at successive at q_2 and q_1, respectively[3].

$$I(q) = \frac{G_2}{q^{s_2}} \exp(\frac{-q^2 R_{g_2}^2}{3-s_2}) \quad \text{for } q \leq q_2$$

$$I(q) = \frac{G_1}{q^{s_1}} \exp(\frac{-q^2 R_{g_1}^2}{3-s_1}) \quad \text{for } q_2 \leq q \leq q_1 \qquad (7)$$

$$I(q) = \frac{D}{q^d} \quad \text{for } q \geq q_1$$

where q is the scattering variable; $I(q)$ is the scattered intensity; R_{g1} and R_{g2} are the radii of gyration for the short and overall size of the scattering object. The parameters s_1 and s_2 helps to model nonspherical objects and d is the Porod exponent. G_1 and G_2 are the Guinier scale factors of intermediate-q and low-q region, respectively; D is the scale factor of the Porod region. To guarantee zero and first order continuity at q_1 and q_2, following equations hold.

$$q_2 = [(s_1 - s_2)/(\frac{2}{3-s_2} R_{g_2}^2 - \frac{2}{3-s_1} R_{g_1}^2)]^{0.5}$$

$$q_1 = \frac{1}{R_{g_1}} [\frac{(d-s_1)(3-s_1)}{2}]^{0.5}$$

$$D = \frac{G_1}{R_{g_1}{}^{(d-s_1)}} \exp[\frac{-(d-s_1)}{2}][\frac{(d-s_1)(3-s_1)}{2}]^{(d-s_1)/2} \qquad (8)$$

$$G_2 = G_1 \exp[-q_2^2/(\frac{R_{g_1}^2}{3-s_1} - \frac{R_{g_2}^2}{3-s_2})] q_2^{2(s_2-s_1)}$$

In case of layered scattering object with thickness T and width W, following equation holds.

$$R_{g_2} = (\frac{T^2}{12} + \frac{W^2}{12})^{0.5}$$

$$R_{g_1} = (\frac{T^2}{12})^{0.5} \qquad (9)$$

$$s_2 = 0 \text{ and } s_1 = 2$$

Eq. (8) can be rewritten as

$$q_2 = [\frac{W^2}{36} - \frac{T^2}{18}]^{-0.5}$$

$$q_1 = (\frac{18(d-2)}{T^2})^{0.5} \quad (10)$$

from which *W* and *T* can be calculated as

$$T = \frac{\sqrt{18(d-2)}}{q_1}$$

$$W = [36\left(\frac{1}{{q_2}^2} + \frac{d-2}{2{q_1}^2}\right)]^{0.5} \quad (11)$$

When q_1 and q_2 are reliably fitted, *W* and *T* can be reasonably estimated.

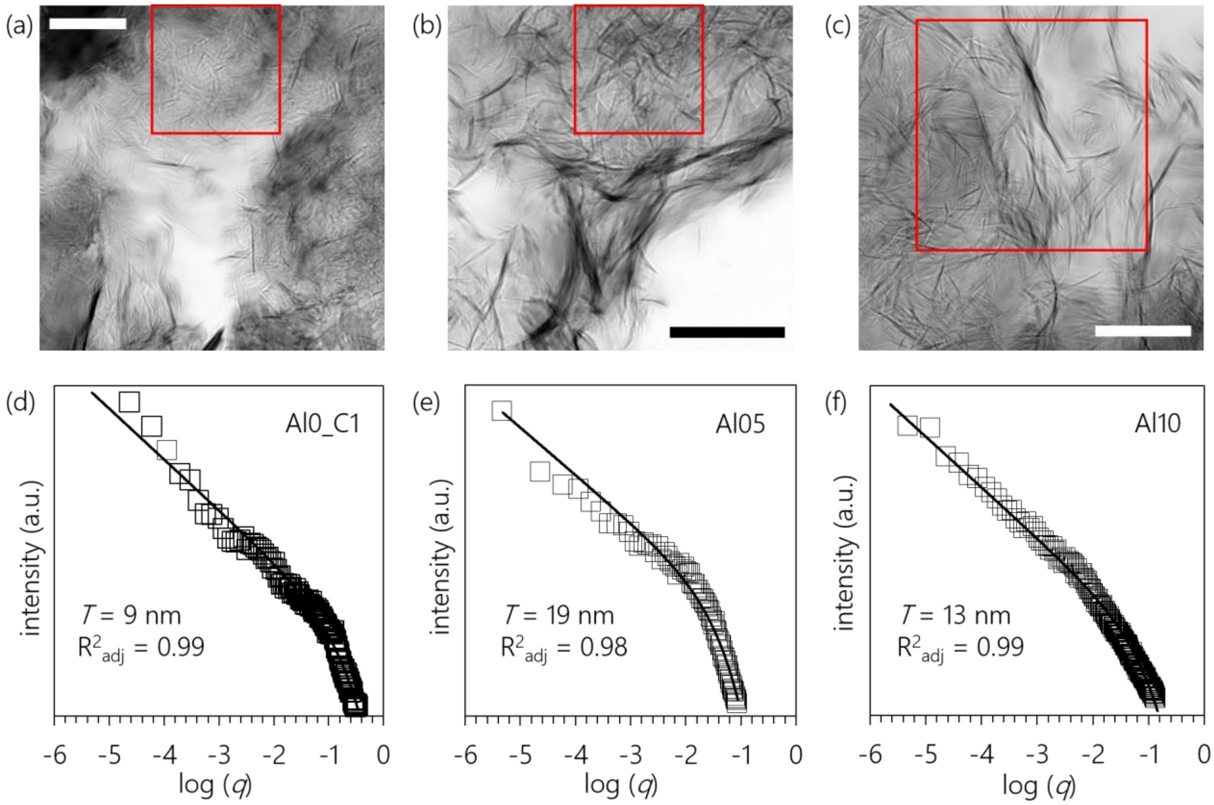

Supplementary Figure 1. Selected areas (red squares) in the ptychographic images of (a) Al0, (b) Al05 and (c) Al10 are subjected to SAS analysis, as shown in (d), (e) and (f), respectively, where black squares are calculated data points and black curves are fitted results. Scale bars are 1 μm. Fitted layer thickness (*T*) and adjusted R^2 of the fitting are indicated in the plots. Estimated standard deviation of the thickness is 2 nm.

In the current work, the calculated SAS has limited extension at low-*q* region, which limits the fitting of q_2 and thus *W*. Only the linear high-*q* region and the exponential region between q_1 and

q_2 are identified. Least square fitting were conducted on q_1, d and G_1 with commercial software (*OriginPro 9*, OriginLab Coporation). The results are shown in Supplementary Figure 1d-f, based on which the thickness T of the layered structure are calculated to be ~ 9, 19 and 13 nm for Al0_C1, Al05 and Al10. Although not being exactly the same with the refined thickness (Table 1 of the main text), the image analysis here does show that the thickness of C-S-H is clearly increased by incorporating Al.

Refining the ambient pressure diffraction pattern of Al05 with different models.

In the current study, the investigated samples are poorly crystalline or nanocrystalline so that the diffractogram has limited number of clearly-resolved peaks. Although such diffractogram does not allow a reliable refinement of the atom positions, it contains sufficient information for refining lattice parameters. To determine the best starting configuration for the refinement, we investigated various tobermorites-based models, e.g. the 11 Å [1] and 14 Å [5] tobermorite models. As suggested by Richardson,[6] there exists a broad range of possible defected-tobermorite configurations. Among the models he proposed, we also selected T_{∞}_11so and T2_so_LS1 as the starting model of refinement, since they have similar unit-cell shape (α=β=90°, γ≈123°, a≈6.7 Å, b≈7.4 Å) as the 11 Å and 14 Å tobermorite models[1-5], which enables a direct comparison of the refined results. Here we use the diffractogram of Al05 at ambient pressure as an example, to compare the refinement using these models (Supplementary Figure 2).

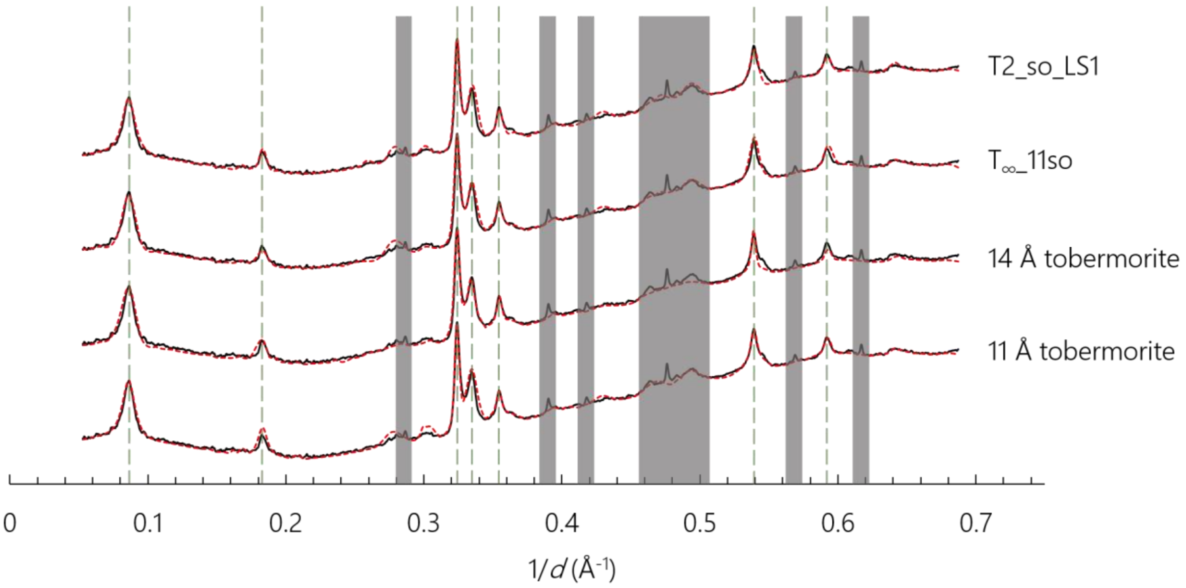

Supplementary Figure 2. The calculated diffractograms (red dashed curves) using the 11 Å tobermorite[1], 14 Å tobermorite[5], T_∞_11so and T2_so_LS1 models[6], plotted together with the experimental diffractogram of Al05 under ambient pressure (black curves). The diffraction peaks of ruby and gasket are masked by grey blocks. The green dashed vertical lines indicate the resolvable diffraction peaks of C-(A-)S-H.

The four investigated models are all able to reproduce the diffractogram of the experimental result, simply by following the refining steps described in the main text (Supplementary Figure 2). As shown in Supplementary Table 1, the refined lattice parameters using different models are highly consistent, with only negligible difference in ***b***. We therefore conclude that the investigated models, although differ in basal spacing, Ca/Si and symmetry type, are all generally able to yield satisfactory refinement. It also confirms that the configurational differences of the studied models cannot be identified by the experimental results. Upon properly refining the lattice parameters, the tobermorite-type structure naturally reproduces the typical C-S-H(I) diffraction pattern[7]. Therefore for consistency among all samples, we conduct all refinement in this study using the same simple model, i.e. the 11 Å tobermorite. The results are shown in the main text.

Supplementary Table 1. Refinement of the lattice parameters (***a***, ***b***, ***c*** and *γ*) of Al05 at ambient conditions using the 11 Å tobermorite[1], 14 Å tobermorite[5], T_{∞}_11so and T2_so_LS1 models[6].

	11 Å tobermorite	14 Å tobermorite	T_{∞}_11so	T2_so_LS1
a (Å)	6.69 ± 0.01	6.69 ± 0.01	6.69± 0.01	6.69± 0.01
b (Å)	7.35 ± 0.01	7.35 ± 0.01	7.34 ± 0.01	7.34 ± 0.01
c (Å)	23.1 ± 0.1	23.1 ± 0.1	23.1 ± 0.1	23.1 ± 0.1
γ	123.0° ± 0.1°	123.0° ± 0.1°	123.0° ± 0.1°	123.0° ± 0.1°

References

1. Brisard, S. *et al*. Morphological quantification of hierarchical geomaterials by X-ray nano-CT bridges the gap from nano to micro length scales. *Am. Mineral.* **97,** 480-483 (2012).
2. Kak, A. C. & Slaney, M. *Principles of Computerized Tomographic Imaging* (IEEE press, 1988).
3. Hammouda, B. A new Guinier-Porod model. *J. Appl. Cryst.* **43,** 716-719 (2010).
4. Merlino, S., Bonaccorsi, E. & Armbruster, T. The real structure of tobermorite 11Å normal and anomalous forms, OD character and polytypic modifications. *Eur. J. Mineral.* **13,** 577-590 (2001).
5. Bonaccorsi, E., Merlino, S. & Kampf, A. R. The crystal structure of tobermorite 14 Å (Plombierite), a C-S-H phase. *J. Am. Ceram. Soc.* **88,** 505–512 (2005).
6. Richardson, I. G. Model structures for C-(A)-S-H (I). *Acta Crystallogr. Sect. B: Struct. Sci.* **70,** 903-923 (2014).
7. Taylor, H. F. W. *Cement chemistry, second ed.* (Thomas Telford 1997).
